# A call for a trauma-informed approach during compulsory care for enduring anorexia nervosa with combined PTSD – an autoethnographic perspective

**DOI:** 10.1186/s40337-025-01283-1

**Published:** 2025-05-27

**Authors:** Jennie Moberg

**Affiliations:** Department of Social Work, Stockholm University, Albanovägen 18, Stockholm, 11419 Sweden

**Keywords:** Anorexia nervosa (AN), Autoethnography, Coercion, Inpatient care, Power threat meaning framework (PTMF), Post-traumatic stress disorder (PTSD), Trauma, Lived experience

## Abstract

**Background:**

Individuals with both anorexia nervosa (AN) and post-traumatic stress disorder (PTSD) often experience significant challenges in psychiatric inpatient care, particularly when coercive measures are used. While the comorbidity of AN and PTSD is well-documented, their interplay in the context of compulsory care and from a lived experience and trauma-informed perspective remains underexplored, despite its critical impact on treatment outcomes. This autoethnographic study aims to shed light on how coercion is experienced in this context, highlighting the need for a trauma-informed approach that acknowledges patients’ histories rather than solely viewing their behaviors as pathological. The Power Threat Meaning Framework (PTMF) provides a theoretical lens for understanding how trauma affects individuals with AN and PTSD, emphasizing the importance of viewing these conditions not just as symptoms to be treated but as survival strategies linked to broader trauma histories.

**Methods:**

An autoethnographic approach was used to analyze blog posts, clinical notes, and diary entries from 2010 to 2019, focusing on experiences of AN, coercion, and trauma.

**Results:**

One overarching theme, ‘Life in inpatient care’, and four subthemes emerged: (1) ‘Being sucked into the vortex of hunger’, (2) ‘Relapse no. 5 and admission procedure – state of emergency’, (3) ‘Encountering coercion and losing control – living in constant fear’, and (4) ‘Managing the aftermaths of eating’.

**Conclusions:**

This autoethnography highlights the interplay between AN and PTSD in compulsory inpatient care, showing how AN served as a coping mechanism for trauma, with starvation as emotional avoidance. The PTMF framework challenges diagnostic-based models, viewing these behaviors as survival strategies rather than pathology. Key findings highlight the impact of coercive treatments such as forced feeding and restraint, which can intensify fear and retraumatization. A trauma-informed approach prioritizing trust, autonomy, and trauma acknowledgment is essential for effective, compassionate care. Recovery should go beyond renourishment to include psychological healing and body-self reintegration, emphasizing a holistic, patient-centered approach for improved treatment outcomes.

**Supplementary Information:**

The online version contains supplementary material available at 10.1186/s40337-025-01283-1.

## Abstract (plain language summary)

The treatment of severe anorexia nervosa (AN) prioritizes life-saving measures, sometimes through coercion such as tube-feeding under restraint. While necessary for survival, these methods risk retraumatizing individuals with post-traumatic stress disorder (PTSD), especially those who use starvation as a coping mechanism. This study applies the Power Threat Meaning Framework (PTMF) as an alternative to symptom-focused diagnostic models, highlighting the role of power, threat, and meaning in shaping distress. Unlike traditional approaches, PTMF considers social and cultural influences on mental health. Given the limited knowledge about the experience of receiving compulsory care for AN and PTSD, this study employs an autoethnographic approach to provide deeper insight into this subject. By drawing on lived experience, it offers valuable perspectives that can help improve trauma-informed and recovery-oriented care in psychiatric settings.

**Supplementary Information:**

The online version contains supplementary material available at 10.1186/s40337-025-01283-1.

## Opening

The staff panicked – why couldn’t she be still? She resisted their commands to rest, and the observers were uncertain about the nature of this behavior, except that it was mistakenly interpreted as sanity. They were at a loss as to how to respond, and she almost laughed at them. They lacked an understanding of starvation or anorexic compulsions, and she remained silent: the inner chanting dictated obedience. Her body was in a state of stasis. *Take your pointless medical examination and get the hell out of here!* The individuals attired in white around the bed lacked an understanding of the significance of unresolved trauma. They were unaware that such trauma could result in a desire to disappear. She smiled discreetly. *Was she really thin enough? Would she ultimately succumb to her afflictions and emerge from them*,* or would she perish in the process?* Subsequently, a doctor entered the room with the IV tubing and a restraint bed. The frail figure before them erupted – spitting, cursing, demanding movement. She was born to run, to jump – *how could she exist otherwise*? At home, prior to the police escort to this godforsaken hospital, she repeatedly evaluated the sharpness of her hips, gripped her thighs with her thumb and forefinger, and, at night, placed one hand over her distressed heart as if to stabilize it. *She never wanted to be touched again*. Now, in a sterile psychiatric ward, she lay rigid, resisting, as if her presence alone unsettled the air. The feeding tube was an instrument of torment – for her and for them. She fought: scratching, spitting, pleading. *Why doesn’t someone press the alarm*? *Why won’t someone save me*? Her own body was subjected to grasping, pulling, and staining, as the fat-filled bodies of others left dirty, calorie-rich prints that would take forever to wash off. For over an hour she sat on the floor of the shower with a female ‘1:1 observator^1^’ who wouldn’t take her eyes off her. “*Fat is contagious*”, she said, crying. The nurse frowned. “*Who would want to exercise in the shower? Don’t you understand that you’re ill?*” The frail figure fixed her gaze upon her and understood that she could never explain her state of mind. Being in control was deeply intoxicating and it had saved her time and time again. So, instead of arguing she kept showering (Diary entry from admission no. 5, week 1, 2014).

## Background

Anorexia nervosa (AN) is a complex condition influenced by biopsychosocial factors, including genetics, unmet psychological needs [1], and personality traits such as perfectionism, rigidity and obsessiveness [2]. It causes significant distress [3–5] and may lead to enduring disability [6]. Despite evidence that AN frequently coexists with trauma [7–10] and post-traumatic stress disorder (PTSD) [11–14], clinical practice might overlook these connections, instead focusing on AN as a disorder of body image disturbance [15]. This risks neglecting the role of childhood trauma in the development and embodiment of AN, as highlighted by Malecki et al. [8], and remains underexplored in treatment approaches [16].

In Sweden, where this autoethnography is set, specialized eating disorder treatment is limited, meaning that many individuals with AN are admitted to general psychiatric wards [17–19]. These wards, rooted in paternalistic traditions [20] can sometimes feel unsafe [21, 22]. In addition, they struggle to implement recovery-oriented care, which in terms of AN prioritizes security, autonomy, and therapeutic relationships [23]. There is limited research on whether these settings help or harm individuals with AN [19, 24]. Given the severe impact of starvation on all organ systems [25], compulsory care – defined as any measure applied “against the patient’s will or in spite of his or her opposition” (26 p. 2) – is sometimes necessary [27–29]. However, compulsory care is often met with anxiety, stress, and resistance [30–32]. Individuals experiencing severe starvation may also exhibit impaired cognition and physical weakness [24], making coercive measures, such as forced feeding and restraint, even more distressing.

Repeated exposure to coercion underscores the intricate and vulnerable nature of enduring AN. The metaphors “walking on a knife’s edge” and “caught between worlds” [33, p. 14] illustrate the fragile balance these individuals navigate, along with those who care for them. While staff focus on survival and renutrition, co-existing conditions such as PTSD from (sexual) trauma [34] are often overlooked. Day et al. [35] further highlight the need for a nuanced approach that considers the impact of trauma and PTSD, emphasizing the necessity of further research to inform evidence-based care. Despite their life-saving intent, coercive interventions – including restraint and tube feeding in a restraint bed – risk reinforcing prior trauma, leading to retraumatization and iatrogenic^2^ impacts [36, 37], where medical treatment unintentionally worsens distress in individuals with AN.

Unlike biomedical models that view AN as a ‘mental disorder/illness’ [38–41], the Power Threat Meaning Framework (PTMF) offers an alternative perspective by stressing the relationship between trauma and AN. The PTMF conceptualizes AN as a response to adverse experiences – a survival mechanism to cope with overwhelming emotions or memories, rather than a pathological condition [42]. PTMF was developed over a five-year period and launched in 2018 [43] as a response to the limitations of medicalized diagnostic models, advocating for a holistic, trauma-informed perspective on distress [44]. Here, trauma-informed care acknowledges the impact of trauma, and fosters safe, supportive environments that promote healing without further harm [45]. Developed collaboratively by psychologists, service users, and activists, PTMF situates distress within social, historical, and cultural contexts [46]. It is structured around four key questions: ‘What has happened to you?’, ‘How did it affect you?’, ‘What sense did you make of it?’, and ‘What did you have to do to survive?’ [44, 46].

As a trauma-informed approach, PTMF shifts focus from diagnosing disorders to understanding the impact of adversities. It validates coping mechanisms as survival strategies, accentuating empowerment, collaboration, and lived experience. In the context of AN, PTMF reinterprets restrictive eating as such a mechanism rather than a disorder symptom. It acknowledges how societal pressures on body image, gender norms, and cultural ideals *intersect* with personal trauma, fostering engagement with one’s history and recovery through empowerment. This approach highlights how behaviors associated with AN, such as food restriction, may serve as strategies to numb distress or create a sense of control in the face of trauma [24, 42]. By providing a framework for exploring how power dynamics, threats, and personal narratives shape an individual’s experience of AN, PTMF offers a more compassionate and holistic lens for understanding eating disorders.

Research highlights a lack of trauma-informed approaches in inpatient mental health services [47] with few studies incorporating lived experiences [48, 49]. To my knowledge, this study is among the first to apply PTMF to AN, and similar to Pellizzer et al. [49], it emphasizes lived experience to inform clinical priorities. Through autoethnography, it explores the AN-PTSD relationship in compulsory care, advocating for compassionate, trauma-informed treatment. Since AN recovery can feel threatening and disrupt coping mechanisms [50], patient-centered care is essential to prevent further distress. By offering an insider’s perspective, this study aims to advance more effective and dignified approaches that acknowledge AN and PTSD’s complexity within inpatient settings.

## Methods

### Autoethnography

Autoethnography challenges conventional research methods [51] by amplifying ‘subjugated knowledge’ [52, 53] and rejecting the objectivity central to positivist paradigms. Instead, it embraces subjectivity, positioning the researcher’s personal experiences as a valid source of knowledge that questions traditional notions of legitimacy in research [54]. Through self-reflection and analysis, autoethnography combines personal narratives with broader social contexts [55], offering what Adams and Manning [56, p. 356] call “insider accounts” – deepening the understanding of subjectivity [57] and uncovering knowledge otherwise difficult to access [58, 59].

Méndez [60] distinguishes between *analytic* autoethnography, which emphasizes objective analysis, and *evocative* autoethnography, which fosters introspection and emotional connection. This study adopts an evocative stance, drawing from blog posts, clinical notes extracts, diary entries, and memories of involuntary psychiatric care. By integrating internal (memories, reflections) and external (clinical notes) data, this approach captures the immediate context of these experiences [61]. Autoethnographic accounts of AN are scarce (see, however, [30, 62, 63]), making this perspective uniquely valuable in understanding compulsory care. It provides: (1) a firsthand account of coercion and loss of autonomy; (2) insight into the internal struggle and ambivalence central to AN; (3) a perspective on care that staff may not fully recognize; (4) a voice to a marginalized group, strengthening their position in healthcare discourse. As Muldtofte [63] notes, the voices of individuals with AN are often dismissed, paradoxically forcing them to “speak through their bodies” (p. 2). Adding to this, and since PTMF encourages personal narratives that foster meaning-making, autoethnography aligns with its core inquiries: ‘What has happened to you?’ and ‘What did you have to do to survive?’ By situating personal experiences within broader contexts, autoethnography not only enhances understanding of AN and PTSD but also solidifies its role as a significant qualitative research method.

### Data: an autoethnographic exploration through blog posts, clinical notes extracts, and diary entries

I have encountered inpatient care (general psychiatric inpatient care and inpatient care specialized in eating disorders) repeatedly since 2004 (hence the ‘enduring’ aspect of AN, [64]). During most of these care episodes, I have frequently written diaries as well as blog posts as a way of documenting events and experiences. Given the focus of this study, I have actively sought for material (blog posts, clinical notes extracts^3^ written by doctors involved in my care, and diary entries) between the years of 2010–2019. The year 2014 was specifically chosen due to the wealth of available material providing a clear and detailed timeline of my experiences, with a well-structured narrative connecting the admission and discharge. The abundance of detail regarding the coercive measures employed and the intertwined anxieties stemming from both AN and PTSD during this period created a particularly rich dataset for exploring the research questions.

Data were initially grouped into key themes that were identified from the texts, such as experiences and/or discussions of AN combined with coercive care, coercive measures, trauma and PTSD. This generated a relatively large body of material, in which I organized blog posts and so on according to the above-mentioned terms. The process of thematizing guided the analysis by identifying patterns within the material. These categories were then refined to reveal overlapping themes and underlying links between personal experience and cultural frameworks surrounding AN and trauma. However, similar to O’Connell [30], I do not regard the data as “direct, unproblematic representations of events” (p. 265). Rather, their value is derived from the insight they provide into the interpretive and meaning-making processes involved in experiences during inpatient care.

### Ethics

An autoethnographic approach offers a nuanced perspective on the ethical dilemmas in compulsory care for individuals with AN, particularly in balancing autonomy and necessary treatment. Ethical considerations permeate the research process, from study design to dissemination, and autoethnography foregrounds the interplay between subjectivity and objectivity, emphasizing self-ethics, relational ethics, and reflexivity [54, 65–67]. Sparkes [54] critiques rigid ethical frameworks, advocating for a context-sensitive approach that navigates challenges like anonymity and confidentiality.

Given the inherent vulnerability in autoethnography [54] I have selectively shared personal experiences while maintaining boundaries, acknowledging that my perspective reflects personal meaning-making rather than an objective truth [30]. Sparkes [54] notes that the act of ‘self-disclosure’ in autoethnography is inherently risky, as it involves the exposure of personal vulnerability [62]. However, similar to O’Connell [30], I would argue that the autoethnography is a public narrative, but due to the aesthetic freedom offered by this methodology, I am able to determine what is presented and what remains unsaid. I have thus selected data that represent challenging periods marked by anxiety and despair, while consciously omitting material that might compromise my integrity (such as detailed information about the traumatic events^4^).

Turning the gaze towards relational ethics means extending beyond the self, shaping how others are portrayed [68]. Because this is an autoethnographic study, consent to participate was not sought. However, with regard to ethical aspects of confidentiality, I have omitted characteristics (such as names, age, appearance etc.) for certain people in order to provide their anonymity.

Lastly, reflexivity is integral to ethnographic research [55, 69], highlighting both risks and benefits of insider knowledge [67]. Berger [67] highlights that an ‘insider’ perspective, while offering familiarity with the subject, may blur ethical boundaries and risk self-absorption, potentially affecting knowledge production. However, lived experience can also provide analytical depth, offering insights that researchers without this perspective may lack, which aligns with the evocative nature of autoethnography.

### Findings

This section provides an interpretative account of my four-and-a-half-month period of compulsory care, where I simultaneously struggled with AN and PTSD. Through interpretations of the autoethnographic data, I explore the dual experience of being both subjected to and reliant on coercive measures, while also seeking understanding and relief from my traumas. My experiences – spanning from my introduction to the ‘anorexic life’ in 2004 to repeated hospitalizations, coercion, and relearning how to eat – are explored through specific extracts, with phenomenological interpretations connecting them. Table 1 provides an overview of the themes.

**Table 1:** An overview of the main theme, subthemes, and their characteristics

Theme name	Description of theme	Example extracts
Main theme: Life in inpatient care	Experiences of inpatient treatment, balancing coercion and recovery.	Coercive practices exacerbated both physical and psychological distress.
Subtheme 1: Being sucked into the vortex of hunger	How starvation became a coping mechanism and an escape from trauma.	By the age of 20, starvation became the answer to everything – intoxicating, soothing, a way to erase my past.
Subtheme 2: Relapse No. 5 and admission procedure – state of emergency	The process of being forcibly hospitalized and the emotions surrounding it.	I remember the police ride, and the overwhelming sense of defeat.
Subtheme 3: Encountering coercion and losing control – living in constant fear	The impact of coercive measures (forced feeding, restraint) on trauma.	Being restrained while fed through a tube reawakened past traumas, making the process unbearable.
Subtheme 4: Managing the aftermaths of eating	The psychological and physical struggles following forced renourishment.	Eating was no longer just about food – it was about fear, and losing control of my body.

### Main theme: life in inpatient care

In essence, the findings highlight the tensions within the care environment, where coercive actions impacted not only my physical health but also my mental well-being, often deepening the traumas I was grappling with. The themes that emerge reflect a life in constant conflict between needing care and feeling trapped in a system that did not always acknowledge or address the underlying traumas driving my anorexic behaviour.

#### Subtheme 1: being sucked into the vortex of hunger

It happened in my early twenties, half a lifetime ago. AN gained strength during periods of excessive running, fueled by my drive to push my limits and my attempt to manage painful memories. By the age of 20, in 2004, I had already become familiar with mental health care due to four years in psychodynamic psychotherapy, as well as being enrolled in adult inpatient care as a consequence of self-harm, suicidal thoughts and behaviour, and PTSD. Starvation and forcing my body to shrink suddenly became a powerful, all-encompassing solution. It was intoxicating and soothing, creating a barrier between me and my past. My nightmares, previously filled with sexually distressing content, were gradually replaced by dreams of food, eating, and hospital weigh-ins.

The compulsive need for control and persistence, deeply ingrained in my personality, became essential to my functioning. Life had always felt like a competition, reinforcing my need for control and achievement. At 16, my psychologist wrote in a journal entry: “J is a young woman who has achieved considerable success in violin playing, writing, and drawing, perhaps to the extent that she has been unable to identify and acknowledge her own emotions”. This turned out to be only part of the explanation for my development of AN. It aligned with my personality and quickly became an addiction unlike anything I had experienced before. I ‘grew’ into it so rapidly that, before I realized it, it was too late to resist.

#### Subtheme 2: relapse no. 5 and admission procedure – state of emergency

I was hungry and cold, but eating and drinking felt like an insult – a sign of defeat. I recall feeling a desire, albeit weak and almost insignificant, to surrender, to allow myself even a *small* bite, perhaps a tomato. Yet, the sense of purity and total control brought a kind of liberation and gratitude: I was free from any thoughts beyond food and the act of not eating. My obsession with self-restraint consumed all my energy, effectively blocking out my past. The rigidity of my condition posed a serious threat to my health. Over time, I became fixated on starvation and its strict rituals. This led me down an unsustainable path, ultimately resulting in another extended stay in inpatient care.

The cycle repeats – this physical state, the unrelenting cold, the entrenched madness. I prepare for my appointment at the eating disorder center, knowing my doctor likely wants to admit me. If she does, it’ll be the fifth time since 2010. But I don’t want to go. I’m too cold, and the thought of someone staring at me – at this misshapen body, this excess fat I believe they see – feels unbearable. AN permeates everything: I mustn’t speak of it or betray it. I feel compelled to freeze, numbing memories and clinging to what remains of my anorexic self. Yet, I go. My doctor, kind but firm, leads me to the examination room as I cry. I want to hide in the basement, to escape the looming threat of another admission. I feel like a two-year-old, refusing to hold cutlery, unable to look at food. I can’t swallow it, I don’t want it! What I do want is to reject it, resist it, avoid it – it’s all part of this twisted game where I’m expected to be grateful but ultimately allowed nothing. Just numbness – eternal peace. (Blog post written prior to admission no. 5, 2014).

This hospitalization began with a phone call from my doctor. We had known each other for five years, and with each passing year, my hospital stays grew longer and more intense: they strengthened my identity of *being* rather than just *having* AN. Days blurred together, filled with nothing but calorie burning and a state of hibernation. I slept deeply, oblivious to my phone buzzing repeatedly beside me. My doctor was trying to reach me, her voice tinged with irritation and worry when I finally answered. The day before, we had stood together in the cold examination room. I had rambled about my fear of water, about not daring to wash my hair because of ‘the calories in the shampoo’. With utmost seriousness, I told her I felt I was ‘consuming’ air, making me fat. She had watched me with concern, reluctantly letting me leave. Now, alarmed by my ‘catastrophic condition’, she wanted to see me again. I was unaware that she had already set a plan in motion – compulsory care was only an hour away. That February afternoon was the coldest I had ever endured. I stamped my feet desperately on the ground, and in a strange emotional haze, I brushed my fingertips against the subway seat without feeling them. I arrived at her door, naive and hopeful, believing I would soon return home and resume my running routine. However, reliance on starvation as a way to manage my memories had taken a severe toll. My vision and hearing were failing, I could barely stand, and my skin had taken on an unnatural yellowish-gray hue. In the examination room, my clothes fell off as my doctor lifted me onto the scales. I interpreted her silence as fear, and her unease strengthened my belief that I was finally becoming anorexic ‘for real’. I proudly described my nighttime runs in the apartment and declared that I no longer slept, believing ‘real anorexics don’t’.

J’s consumed by an intense fear of calories and delusional beliefs about her body. Her ability to communicate is impaired, and she’s trapped in the impossibility of increasing her food intake. When informed of the need to eat, she becomes visibly distressed, threatening to damage feeding tubes and induce vomiting if hospitalized for tube feeding. Due to the somatic danger posed by continued starvation, compulsory care is now necessary. I’ve issued a compulsory care certificate and requested police assistance to transport J to the ER. Outpatient care is no longer viable, as J refuses voluntary treatment. She suffers from severe and enduring AN and has been referred for PTSD treatment but has not yet begun therapy. J lacks insight into the life-threatening nature of her condition, repeatedly insisting on being allowed to exercise and expressing panic over sleeping, which she associates with a decrease in metabolic rate. Given her fragile state, I believe it’s highly inappropriate for J to be active during inpatient care. I recommend 24/7 monitoring, strict bed rest, and the use of a wheelchair for any necessary movement. (Clinical notes extract written prior to admission no. 5, 2014).

‘Compulsory care certificate’. Those words echoed in my mind as her calm voice continued, ‘The police are coming, Jennie. You need to go to the hospital’. I stared blankly at her moving lips before collapsing into her arms. Suddenly, panic overtook me. I spat, hissed, and kicked until I broke free from her grip. Then I ran. She went after me, showing clear signs of distress. Our footsteps echoed in the corridors and she called for help from passing colleagues. I remember her quick intake of breath when she finally got hold of my arms and pressed herself close to me, holding me. Due to fatigue, I then passed out in her room, and woke up with my head in her lap. ‘Your body’s at its limit. I fear you could die at any moment’. I was placed in the back of a police car, and a wave of anxiety washed over me. The inpatient care staff would make me gain weight, undo everything I had achieved, and take away the only things that eased my pain. I would not be able to run, eat my strict X-calorie diet, or be left alone – I knew the drill. I glimpsed the hospital’s imposing entrance from the corner of my eye. The policemen lifted me into a wheelchair as my doctor led the way into the ER. In the car, she had taken my hand and squeezed it in a gentle manner. I could tell she did not really want to do this, but she had no choice. After two nights in cardiololy, where potassium deficiency was corrected and the ongoing arrhythmias were resolved, I was transferred to the familiar psychiatric ward by ambulance. As the door opened, I was greeted by a familiar voice: ‘Are you here again, Jennie?’ and gently guided into bed. There I lay, exhausted, clinging to an irrational pride in barely existing.

#### Subtheme 3: encountering coercion and losing control – living in constant fear

The day after my admission, my doctor called, but I could not bring myself to speak with her. I was conflicted, torn between gratitude for her intervention and resentment for the same reason. A fellow patient drew a picture of me, bluntly commenting on how ‘unattractive’ I looked, suggesting I must have appeared ‘much better without AN’. I responded that beauty was not my concern – I had more pressing issues than being viewed as sexual material or mere female flesh. Indeed, my primary struggle was managing the overwhelming panic of being forced to abandon AN. This panic was all-encompassing – invading my dreams, lingering when I woke, following me in the wheelchair, down the corridor, and even into the shower. I could not bear the thought of my body changing, slowly softening due to proper nutrition. Food became inescapable. Whether in solid form or as liquids through the nasogastric tube, it was impossible to avoid. This triggered fear that faded only when the perceived danger lessened. But on the ward, the danger never disappeared, it fed on itself. I began hiding food, even under the constant watch of the staff. Somehow, I managed to fill my bed with cheese, bread, potatoes, and other absurdities. Ironically, I had built a makeshift pantry around my own body, all while under their unblinking gaze.

A male staff member offers me a piece of leftover cake from their meeting room, citing its ‘high calorie content’ as a way to ‘avoid tube feeding’. *Prick*. The irony is palpable. I can’t eat, and I struggle to remember when I last dared to eat cake – maybe at 18? The absurdity of this situation ignites a sudden, intense anger in me. I hurl a coffee cup to the floor. Inexplicably, someone hits the alarm button over spilled coffee (!) and my outburst. A piercing sound fills the air, and chaos begins. Staff enter my room, subduing and lifting me onto the bed. My hair is in my eyes, my body aches, and I feel like a piece of meat being split open. As they restrain me and insert the feeding tube into my nose, I shake my head and yell, ‘*Don’t touch me!*’ The invasive procedures are devastating – it feels like dying as tubes and fluids are forced into my body. Flashbacks overwhelm me. Lying there, I’m acutely aware of the anorexic losses and calories flowing down my throat. I feel exposed, as if under a microscope, with my limbs splayed out. In despair, I cry out: I hope you all think this is worth it, because you’re fucking destroying me! (Diary entry from admission no. 5, week 2, 2014).

Life in inpatient care felt like existing in a bubble – detached from reality, defined by a cycle of sleeping, ‘eating’, hesitating, hoping, and parrying. The never-ending corridor laying there, fixed and static. My outpatient doctor visited, her presence a reminder of the life I was not living. In our conversations, AN did not stand a chance. She attacked its very essence while still validating my anger, fear, and despair. She listened to my beliefs but skillfully dismantled them. Despite my struggles with recurring trauma, she managed to instill hope.

During a conversation, shortly after having been force-fed yet again, she settled into the armchair in my room. I looked at her from the outpost in my bed, resting my hands on the velvety blanket my youngest brother had bought me. We discussed the present, past, and future, despite its uncertainties. She encouraged, no, she *begged* me not to give in to AN completely. She even challenged me to accept my past, cruel as it was. In doing so, she asked me to relinquish the control I had clung to so desperately for so long. This meant confronting the reasons behind my decision to resort to starvation. She now stepped into mined territory, contributing to me holding my breath in anger before starting to talk about trauma instilled memories. Discussing this was an inherently painful process.

J, still cachectic, shares her traumatic childhood experiences with me. She expresses disgust at being ‘filled’ and ‘invaded’ by food during meals, drawing parallels between these sensations and her past traumas. She sometimes views her self-starvation as a misguided protest against her childhood abusers, acknowledging that her AN is largely fueled by anger. When discussing these complex memories, J’s facial expression often becomes weary, and she occasionally appears unresponsive due to flashbacks. She denies hearing voices, other than the anorexic one, and admits to feeling that things are sometimes ‘unreal’. However, she recognizes this as a feeling rather than reality, indicating varying degrees of dissociation. J reports experiencing frequent flashbacks and heightened anxiety due to repeated force-feeding. We discuss how her memories will persist regardless of whether she starves or eats, emphasizing the need for her to learn to manage these memories without relying on AN. (Clinical notes extract from admission no. 5, week 5, 2014).

One day during a blood test, a nurse casually mentioned that when the compulsory care certificate was written, I had been near death. My prognosis had been terminal, with only two days left before I would have succumbed to starvation. Then, as if discussing something routine, she asked if I was interested in antipsychotic medication to control my anorexic ‘voice’. If so, she would pass it on to the doctor. The idea of being so close to death was incomprehensible – I had never considered myself a ‘true’ anorexic. Instead, I wanted to explain that the sense of control and euphoria I experienced was so powerful and intoxicating that I was willing to do anything to maintain it. The realization that the staff were stripping me of everything became a monolithic, cyclical thought. Their actions, such as repeatedly lifting me to the restraint bed, left bruises on my arms and legs and felt retraumatizing. All I wanted was to sleep, or at least hide under a mountain of blankets. I must have dozed off because I was suddenly jolted awake by staff entering my room, interrogating me about the calories I had or had not consumed. *As if I was to tell them?!* I hesitated, and before I could fabricate a lie, I was restrained and prepped for nasogastric tube insertion. The experience left me in shock. I tried to shut down my consciousness to cope, but the pain of my stomach expanding from tube feeding made it impossible. An overwhelming sense of unease gripped me, one that even intravenous sedatives could not mitigate. After this subsequent and severe anxiety attack, I spoke with the medical director whom I had known and trusted for years. Aware of my trauma history, she redesigned future coercive procedures using a trauma-informed approach. This approach emphasized collaboration and minimizing unnecessary distress, and was documented in my clinical notes for staff to adhere. After this, being subject to coercion made the situation not in any way ideal, but quite different.

J, a woman with severe AN, remains in a life-threatening state of starvation. Due to insufficient nutrition intake today, tube feeding has been deemed necessary. J was given the opportunity to eat and drink independently but was unable to do so due to severe anxiety. Despite a history of cardiac arrest and recent arrhythmias resulting from AN, J strongly denies the life-threatening nature of her condition. Her cardiac status is closely monitored through regular sampling and ECG examinations. To minimize J’s discomfort, she’ll be placed in the restraint bed calmly and safely, without staff using excessive force to hold or grab her. The bed’s head will be raised to allow her to observe her surroundings and the staff in the room, which may help reduce feelings of helplessness. Before initiating restraint and renutrition, I will carefully explain the procedure to J, ensuring she understands what will happen. Once informed, the feeding tube will be inserted, and tube feeding will begin. Afterward, I will follow up with J to assess her response and provide support. This approach seeks to address J’s critical medical needs while prioritizing her emotional well-being during this difficult intervention. (Clinical notes extract from admission no. 5, week 7, 2014).

#### Subtheme 4: managing the aftermaths of eating

With the increased nutritional intake, the flashbacks intensified. Only by forcing my body into a state of non-existing could reduce them. Dealing with the aftermaths of eating was a step-by-step process. During inpatient care, it began with an acute phase marked by intense fear and avoidance of food. This was followed by stages where new eating habits were (forcibly) introduced. As my physical health improved, it became possible to address psychological issues. In these later phases, I started to crave external stimulation, something I could not handle during the acute stage. Prior to admission I could not work or study, and had lost interest in activities I once loved, such as playing the violin, reading, playing chess, drawing, and writing. Survival became my sole focus, making it nearly impossible to confront or process traumatic memories. After an initial period of discord and misunderstanding with the staff, I gradually attempted to eat more with their support, and a fragile ‘truce’ developed. During this phase, my need for both emotional and practical support – from staff and loved ones – grew stronger. Reaching out to important people in my life provided a sense of comfort and stability. It was essential to maintain this truce while working to rebuild the trust that had been severely strained in previous care experiences. More than anything, I needed to be reminded of the power of kindness and empathy.

I’m told that I need to relearn to chew and swallow, skills I once had. But eating makes me feel swollen, angry, and enormous. It’s repulsive because it reminds me of my body. This feels overwhelming. I cry, knowing I must endure it, yet having no idea *how* I’ll manage. To the sweet and funny staff member who accompanies me during mealtime I say ‘The past, present and future intersect. Everything’s connected. The suffering from then is the suffering now’. Her wise, youthful gaze makes it impossible for me to attempt escaping through the locked door. ‘Do you want to throw some eggs?’ she suddenly asks. Red-faced from sobbing, I nod after a moments pause. I grab my black blanket before she wheels me to the hospital entrance. In my hands lie three brilliant white eggs, feeling like precious gifts I must protect. The fresh air hits me – I’ve been inside for months. It’s 20:08. I take aim and throw the eggs at a tree, one by one, screaming. It feels incredibly good to express anger. We laugh together. In the elevator, returning to the ward, I cover my thighs with the blanket. I’m amazed I got to do this – I’ve found my anger again. It’s as if she knew *exactly* what she was doing when she gave me those damn eggs. (Blog post from admission no. 5, week 11, 2014).

After about three months of treatment, I began to truly grasp how close I had come to dying – again. Yet, trying to suppress AN felt futile: it always found its way back. In truth, since 2004, it had never really left me. Instead, I had nurtured it, drawn to the sense of freedom it gave me, both in being controlled and in controlling my femininity. But standing before the ward mirror with a clearer mind, no longer clouded by the ‘I’m soon going to die’ mentality, I occasionally saw myself for what I had become: a worn, hollow figure with sunken cheeks and bulging eyes. My skin was dry and brittle, my hands gnarled, my shoulders painfully sharp. I felt a sense of satisfaction in not looking like a typical woman. My body no longer reflected traditional femininity, and in that, I found relief. Yet, the realization that I could not keep my body in this exact state sent me into a desperate cry. Later, I stared out the third-floor window before asking the staff member monitoring me to escort me into the corridor for coffee. When we returned, we had a long conversation about life. She reminded me of my long-term goals – like pursuing academia – while I watched the leaves of the ivy beside my bed, a gift from my therapist. That evening, the staff and I played chess, a distraction from the nutritional drink I had to finish to avoid further tube feeding. During the late hours, a female carer let me sit in the hallway, wrapped in blankets with a cup of tea in my hands, watching her bake bread. This quiet ritual soon became essential, helping me prepare for the stillness of the night and keeping the nightmares at bay. Her presence, so simple yet kind, was something I quickly clung to. The next day, visitors would come. Maybe I would even go outside. Was I finally beginning to settle? Could I actually survive this?

Mom and Dad visit me in the hospital. Their eyes flit between the peripheral vein catheter in my forearm and the IV pole by my bed, a mix of fear and relief in their gaze. They bring a bag of personal items: my favorite perfume, clothes, pinstriped coat, bills, diet soda, poetry, and a hair tie. As we huddle together, something breaks inside me. I look at them, acutely aware of what I might lose. In here, life’s a problem to be solved. Out there are opportunities, pure thoughts, people breathing freely. Through the hospital window, I hear seagulls squawking between my tears and racing heartbeats. Swallowing tranquilizers before a meal that includes rice, I think, ‘This can’t go on forever. Someday AN will have to leave me voluntarily – without being forced’. With my parents in the room, we define hope differently. They wish for my full recovery, while I pray for a life manageable despite lingering anorexic thoughts, behaviors, and impulses. We sip coffee, attempting smiles I’ve almost forgotten how to form. Before they leave I tell them that I’ll try my best not to be afraid. I promise them that I’ll live. (Blog post from admission no. 5, week 13, 2014).

## Discussion

This autoethnography sought to explore my lived experience of AN and PTSD within compulsory inpatient care. The study aimed to highlight how these conditions were intertwined, and through this exploration, it showed the complex relationship between them, revealing their profound psychological impact. The argument was made that compulsory care should be provided in a manner that is both effective and compassionate. The study also addressed how coercive medical practices – though lifesaving [29] – retraumatized me when not applied with sensitivity to my underlying emotional needs. During the admission, it was stated that my prognosis had been ‘terminal’, and this concept, while contentious and contested by Gaudiani et al. [70], raised crucial questions about the limitations of traditional treatment approaches. My experience suggested that a ‘terminal’ prognosis may have been premature if trauma-informed and person-centered strategies, as outlined in the PTMF [44, 46], were not fully explored. By framing AN as a response to trauma and power imbalances, the PTMF offers an alternative lens that emphasizes understanding and meaning-making over symptom reduction, extending beyond mere survival. However, my experience of forced feeding and restraint exemplified how compulsory care, if not permeated by collaboration and delivered with empathy, could heighten fear and helplessness while alienating patients from the therapeutic process. By incorporating PTMF [44, 46, 47], this study challenges the notion of AN and PTSD behaviors as mere symptoms of illness, instead framing them as coping strategies developed in response to trauma [71]. This perspective shifts the focus from disorder to lived experience, fostering a more compassionate and effective approach to care. Recognizing distress as a meaningful survival response and actively involving patients in treatment could significantly aid recovery. This aligns with lived experience literature on trauma-informed care and shared decision-making, underscoring the importance of validating personal histories in clinical interventions [48, 57]. Pellizzer et al. [49] further emphasize the significance of incorporating lived experience perspectives in eating disorder research, advocating for person-centered and adaptive approaches that prioritize individualized care and recovery-oriented outcomes. For me, this meant care that acknowledged the underlying trauma driving my distress rather than focusing solely on refeeding and weight restoration. A truly patient-centered approach thus involved fostering trust, minimizing coercion where possible [29], and incorporating my voice in decision-making, which aligns with trauma-informed principles that facilitate recovery pathways [71].

### The interplay of AN and trauma

It is important to acknowledge that the concepts of control and AN as a form of psychological sanctuary are well established in the literature on AN [72]. While this study applies PTMF to reinterpret such themes within a trauma-informed context, the intention is not to suggest that these insights are novel. Rather, PTMF serves to revisit and recontextualize them, linking them explicitly to experiences of power, threat, and survival.

One of the major insights from this autoethnography is the complex relationship between AN and trauma. For me, AN functioned as a coping mechanism – an attempt to manage and suppress memories of past trauma, including self-harm and suicidal thoughts stemming from the trauma itself. On the ward, staff were expected to monitor and correct ‘dysfunctional’ anorexic behaviors and thought patterns, which, however, undermined my trust in them and led to resistance when I was no longer allowed to starve. As such, starvation had helped me to control thoughts, as well as my body and, in doing so, distance myself from emotional pain. This aligns with existing research, such as Bryant et al. [42], which highlight the varied functional aspects of AN, including its role as a survival strategy for trauma. My experience further supported the findings of Kiely et al.’s meta-synthesis [24], which detail the treatment needs of individuals with severe and enduring AN. Similar to findings in that study, I found that inpatient care primarily focused on renourishment and weight restoration while neglecting the emotional and psychological aspects of my distress. This echoes the critique in Kiely et al. [24], where treatment for SE-AN is described as being narrowly centered on medical variables such as body weight, while neglecting the broader, person-centered needs essential for recovery. In my case, eating and nourishment were not merely physical acts but psychological burdens, perceived as threatening, defeating or insulting as well as evoking negative emotions [73] such as shame and guilt. Medical interventions, though necessary, often exacerbated my distress by overlooking the trauma underpinning my AN. When treatment was dictated by diagnosis alone, behaviors like starvation were more easily dismissed as symptoms rather than as meaningful attempts to cope [44, 46]. My experience of inpatient care felt largely mechanical, aside from interactions with certain staff members who saw me as a person rather than just an ‘anorexic’^5^. Because the primary focus was on renourishment, I was left feeling isolated and unable to fully engage in learning to eat without feeling dirty or wanting to die as a result.

### The role of coercion in treatment

Compulsory care, sometimes recognised as necessary for life-saving treatment [74], should preferably be implemented with sensitivity. Studies on patient perspectives of psychiatric inpatient wards indicate that they value staff who engage in meaningful communication and foster therapeutic relationships [22]. Failing to implement coercive measures with dignity, in turn, risks harm these relationships. For me, involuntary hospitalization, forced feeding, and restraint were traumatic experiences that triggered intense feelings of fear and helplessness, reinforcing bodily memories of past trauma. While my account includes the use of physical restraint, it is important to acknowledge that the prevalence and application of such measures may vary across countries and clinical cultures. Physical restraint is not always used in the treatment of severe AN [75]. My experience should therefore be understood in light of these cultural and systemic differences, and not as representative of standard practice globally. Although these interventions were life-saving [29], they indeed increased stress and ambivalence – reactions that have been frequently described in qualitative accounts of individuals with AN [30] undergoing involuntary treatment [76]. While staff often acted out of genuine concern and adhered to established medical protocols, it is important to consider the psychological impact their actions had on me. A more trauma-informed approach could have involved recognizing this emotional toll and adapting care strategies to reflect a deeper understanding of trauma and its effects. This aligns with PTMF principles [44, 46, 47], suggesting that power imbalances in psychiatric care risk perpetuating distress rather than alleviating it. This alternative approach, grounded in acknowledging individuals’ histories and fostering safety and trust, offers a pathway for healthcare providers to mitigate the psychological harm of coercion. By recognizing the impact of power dynamics and prioritizing patient experiences, clinicians could provide a more supportive and effective care environment. Validating distress, integrating empathy, and recognizing AN as more than a disorder of weight and food could have transformed my experience. Moreover, a trauma-informed approach such as the PTMF fostered therapeutic relationships based on trust and respect [76, 77]. As such, shifting away from paternalistic and rigid routines enabled a more collaborative approach that prioritized hearing and valuing the patient’s voice. Although this approach may require more time and effort, I, similar to Asaria [78], believe it to be essential for building a positive therapeutic alliance and ensuring that care goes beyond survival to genuine recovery.

A central theme during inpatient care was my battle for autonomy [77]. Prior to the admission, extreme food restriction had given me a sense of control when everything else felt chaotic. Forced renourishment violated my integrity, intensifying feelings of failure and self-hate while, as described by Muldtofte [63], also resulting in a loss of subjectivity. I would argue that the challenge here lay in finding equilibrium between clinical intervention and honoring individual autonomy, and this study has outlined how such a balance might be achieved. Integrating lived experience into treatment planning and prioritizing trust-building are essential strategies for effectively navigating the intersection of AN and PTSD. Approaches that focus on minimizing retraumatization and reducing iatrogenic harm [30, 36, 37], offer a pathway toward more collaborative and person-centered care for complex trauma-related conditions, supporting both effective treatment and respect for personal choice.

### Embodiment in anorexia nervosa

Being adamant about avoiding food was, for me, not just about refusing the food itself, but also about refusing memories and the actual embodiment of them – that is, *my* body. By doing so, I may unintentionally have contributed to the ‘objectification’ of myself. Similar to O’Connell [30], I wanted to embody my condition, constantly monitoring myself to ensure I was ‘doing’ AN correctly and successfully. Malecki et al. [8] challenge the traditional body-image paradigm in understanding AN [15], particularly for women with histories of childhood trauma. Rather than viewing AN solely as a disorder of body-image disturbance (as mentioned in the introduction), they highlight how it is deeply rooted in disembodiment – the experience of one’s body as an object to be controlled rather than a lived reality. For many women with trauma histories, myself included, the body became something external, detached from the self [8], as opposed to a source of agency and presence. This estrangement was manifested through restrictive behaviors, which served as a means of exerting control [7] and creating distance from painful emotions. In my own experience, starvation was not just about weight – it was a way to *disconnect* from and dominate my body, as well as to suppress memories that felt too overwhelming to confront. Medical interventions, particularly forced feeding, intensified this disconnection. Rather than facilitating a reconnection with my body, these measures reinforced the idea that my body was something to be acted upon by others, further stripping me of dignity and autonomy. Malecki et al. [8] argue that traditional clinical approaches fail to capture this complexity, reducing women’s experiences to measurable distortions while overlooking the intricate interplay between trauma, embodiment, and survival. Their research advocate for a more nuanced and comprehensive understanding of AN – one that surpasses pathology and recognizes the body as a space of both suffering and resilience. As shown in the findings section, the reasoning by Malecki et al. [8] could be applied to the doctor who, in the clinical notes, ‘acknowledged’ the distress caused by repeated tube feedings, leading to reconnection with past trauma. In turn, this acknowledgment also recognized the nature of the trauma and the power dynamics between PTSD and the perpetuation of AN.

### Strenghts and limitations

This autoethnographic study offers a unique and deeply personal perspective on complexities of AN and co-occurring PTSD in compulsory psychiatric care. One of its primary strengths is its ability to provide an authentic, firsthand account of the lived experience of involuntary treatment [59]. By situating personal experiences within broader cultural and clinical contexts, this method captures emotional and psychological struggles that may be difficult to access through traditional research approaches. The study highlights the vulnerable and often invisible experiences of patients, shedding light on the impact of coercion, trauma, and loss of autonomy in inpatient care.

However, the subjective nature of autoethnography introduces potential bias and challenges to generalizability. Since the findings are based on personal experience, they may not fully reflect the diverse range of experiences among individuals with AN and PTSD. Nevertheless, this limitation is mitigated by aligning personal narratives with existing literature [30, 58], reinforcing the validity of the insights shared. Additionally, the personal nature of autoethnography raises ethical concerns. While self-disclosure carries inherent risks, including emotional distress [66], it can also serve as a tool for meaning-making and reflection. To address concerns regarding subjectivity, reflexivity was applied throughout the study, ensuring that personal insights were critically examined within the framework of trauma-informed care and psychiatric treatment. Despite these limitations, this study demonstrates how lived experience can contribute to scholarly discourse, offering a nuanced, patient-centered perspective that challenges conventional treatment models. By integrating personal narratives with trauma-informed frameworks [44, 46], it provides valuable insights into how psychiatric care can evolve to better address the needs of individuals with AN and PTSD, ensuring that medical interventions are balanced with a commitment to psychological safety.

## Conclusions and implications for practice

This autoethnography highlights the need for a trauma-informed approach in the compulsory care of individuals with co-occurring AN and PTSD. While life-saving interventions such as forced-feeding and restraint may be necessary in extreme cases [29], their implementation must be adapted to minimize retraumatization and distress. A key message from this study is that psychiatric care should move beyond a purely medical stabilization approach to incorporate psychological and emotional healing. To improve treatment experiences and outcomes, integrating trauma-informed principles among clinicians in inpatient care seems essential. One such principle addresses trust as a core component, and is fostered through transparent communication. Clearly explaining the necessity of interventions and actively involving patients in decision-making where possible can help reduce fear and resistance. Further, prioritizing patient autonomy, even within a compulsory care context, is crucial. Allowing individuals some degree of choice - whether in meal planning, the selection of coping strategies, or determining who is present during nasogastric feeding (with the ability to exclude male staff if desired) – can help mitigate feelings of powerlessness. Recognizing that AN may function as a coping mechanism for trauma [44, 46, 47] is also vital in shaping compassionate and effective care strategies. In addition, minimizing unnecessary coercion should be a central focus in treatment. Implementing de-escalation techniques, avoiding punitive responses to distress [77], and exploring alternatives to physical restraint [76] whenever possible can significantly reduce (iatrogenic) harm [36, 37]. Furthermore, adopting a holistic perspective that integrates both the physical and psychological aspects of recovery is necessary. Addressing body awareness and self-compassion within treatment can help individuals reconnect with their bodies, shifting the focus from rigid renourishment protocols to interventions that foster safety and long-term healing [8]. Inpatient settings should acknowledge that many individuals with AN, particularly those with trauma histories, experience their bodies as objects to control rather than as intrinsic parts of themselves. By addressing these factors, psychiatric inpatient care can evolve beyond mere medical stabilization to adopt a more ethical and patient-centered framework.

### Ending

Is it really over? Did I make it? After four and a half months of an unrelenting battle, today I’m being discharged. When I arrived 131 days ago, it was winter: now, summer has arrived, and with it, thousands of thoughts have passed through my mind. I’m stepping out into a wider world, a greater freedom – yet, through counting calories and avoiding sitting down, I’m not truly free at all. But after everything, I’m still here. Still breathing. Still alive. If someone were to ask me who I am, I wouldn’t know how to answer. All I know is that I’m no longer who I once was, and perhaps I never will be again. Maybe I’ll never fully heal, or perhaps healing’s just another transformation – one I’ve yet to understand (Blog post written upon discharge from admission no. 5, week 18, 2014).

## Electronic supplementary material

Below is the link to the electronic supplementary material.

Supplementary Material 1

## Data Availability

No datasets were generated or analysed during the current study.

## Footnotes

1 Also known as ‘constant observation’ or ‘constant supervision’ where staff monitor patients 24/7 in order to prevent suicide or serious self-harm. With regard to AN, self-harm could manifest in obsessive physical activity, and/or self-induced vomiting.

2 Iatrogenic refers to a condition or illness that is induced unintentionally as a result of medical treatment or the actions of healthcare professionals.

3 In Sweden, to access clinical notes as a patient, you generally need to request them from the healthcare provider (hospital/clinic). They are legally obligated to provide them, with some limited exceptions (e.g., if disclosure would cause serious harm to you or someone else).

4 A positionality statement is available on request as a separate file for readers who wish to gain a more in-depth understanding of the researcher’s background and perspectives.

5 These interactions are understood as ‘helping’ and are an important aspect of recovery research. However, these relationships are not my primary focus for this study, and must be explored in greater depth in a different context.
