# Supplementary material for: A call for a trauma-informed approach during compulsory care for enduring anorexia nervosa with combined PTSD – an autoethnographic perspective
Source: J Eat Disord. 2025 May 27;13:92. doi: 10.1186/s40337-025-01283-1 (PMC12107862; doi:10.1186/s40337-025-01283-1)
Supplement: Supplementary file 1 — Supplementary Material 1 [file 40337_2025_1283_MOESM1_ESM.pdf]

Positionality and reflexivity statement

As the author of this autoethnographic study, I recognize that my personal experiences of trauma, enduring anorexia nervosa (AN), post-traumatic stress disorder (PTSD), and several years in psychiatric inpatient care inevitably shape the lens through which I approach this work. This research is deeply intertwined with my lived experiences, providing both a unique depth of understanding and a necessity for ongoing reflexivity.

Lived experience and research perspective

My struggles with AN and PTSD stem from sexual abuse in early childhood, which I, through intrusive flashbacks, became more consciously aware of by the age of 11. The human cost of such trauma is profound, encompassing years of physical and emotional suffering due to various self-destructive behaviors, strained relationships, and the loss of opportunities for growth and well-being. These experiences shape not only my personal journey but also my academic inquiry into the intersection of trauma, mental health care, and patient autonomy.

While my lived experience provides a unique and deeply personal insight into the intersection of AN, PTSD, and coercion, I acknowledge the potential for bias. My positionality as both a patient and a researcher necessitates careful reflexivity to ensure that my interpretations remain grounded in empirical evidence rather than personal grievances or uncritical advocacy. To mitigate this, I have engaged with diverse perspectives, integrating existing literature and theoretical frameworks, particularly the Power Threat Meaning Framework (PTMF), to contextualize my findings within broader discourses on mental health and trauma.

Timing of trauma work in treatment: A critical reflection

The prevailing clinical assumption that trauma-focused therapy should begin only after nutritional stabilization often overlooks the reality that, for many, the eating disorder itself is a response to trauma. Coercive care, though sometimes medically necessary, risks addressing only the immediate physical crisis while neglecting underlying psychological issues, exacerbating anxiety and distrust in the care system. Delaying trauma work may further entrench ‘disordered behaviors’ – a perspective my experiences strongly reinforce.

During my treatment, AN functioned as both a coping mechanism and a response to past trauma, with starvation providing a sense of control over internal chaos. Forced interventions, such as restraint and nasogastric feeding, triggered deep distress, evoking past trauma and a profound sense of powerlessness. As reflected in my autoethnographic account, the quote, *"She now stepped into mined territory, contributing to me holding my breath in anger before starting to talk about trauma-instilled memories"* encapsulates the emotional dissonance that arises when trauma is addressed in an environment of coercion rather than collaboration.

A significant debate in the literature concerns the timing of trauma-focused therapy in AN treatment. While conventional wisdom suggests PTSD treatment should only begin post-stabilization for psychological reasons, this perspective often fails to recognize that for some, AN itself is a *manifestation* of trauma. My experiences challenge the notion that trauma work must wait until after weight restoration, highlighting a critical gap: if trauma is not acknowledged throughout treatment, compulsory interventions risk being retraumatizing rather than restorative.

Ongoing treatment and recovery

While this study focuses on past experiences, my treatment journey continues. At the time of writing, I am still struggling with AN, and it remains a constant presence in my life, greatly restricting me in various contexts. In 2016, I stopped pursuing 'full' (clinical) recovery. Since 2017, I have been periodically enrolled in an outpatient clinic that specializes in supporting individuals with enduring eating disorders where previous treatments have not achieved the desired or long-lasting effects. This clinic prioritizes quality of life improvements and has provided the support needed to find balance while also fostering my academic journey as a PhD student.

An attempt was made to address my traumas using 'Eye Movement Desensitization and Reprocessing' (EMDR) therapy in 2004, but this caused more harm than good due to inadequate preparation and stabilization techniques, which are crucial before initiating such therapy. At the time, I was not able to exercise any coping skills beyond starvation and self-harming behaviors to manage the anxiety that such therapy often entails. However, after having underwent 'Prolonged Exposure Therapy' 2015–2016, I have made progress in managing my PTSD. In essence, I believe this ongoing engagement with treatment

allows for a dynamic, evolving perspective on recovery – one that is not linear but instead fluctuates.

Importantly, continued treatment has also allowed me to critically reflect on the experiences documented in this study. The process of integrating trauma-informed approaches into my care has further reinforced the argument that recovery must extend beyond renourishment – a perspective central to this research. While weight restoration remains a necessary component of treatment, true healing involves psychological safety, trust, and the re-establishment of autonomy, elements that were often missing in earlier interventions.

Conclusion

This statement serves as an acknowledgment of the ways in which my past informs my research and my commitment to transparency in presenting both subjective and analytical insights. By explicitly situating my experience within this study, I hope to foster critical engagement with the ethical, clinical, and philosophical dimensions of compulsory care. Ultimately, I advocate for a more compassionate, trauma-informed approach that recognizes the complexities of AN and PTSD beyond weight-centric models of treatment.
